# Supplementary material for: The Systems Biology Research Tool: evolvable open-source software
Source: BMC Syst Biol. 2008 Jun 29;2:55. doi: 10.1186/1752-0509-2-55 (PMC2446383; doi:10.1186/1752-0509-2-55)
Supplement: Additional file 1 — SBRT Archive. An archive of the current version of the Systems Biology Research Tool. [file 1752-0509-2-55-S1.zip › sbrt-1.4.0/doc/developers_guide/api/sbrt/shell/io/class-use/OutputFile.html]

Uses of Interface sbrt.shell.io.OutputFile


|  |  |  |
| --- | --- | --- |
| **Overview** \| **Package** \| **Class** \| **Use** \| **Tree** \| **Deprecated** \| **Index** \| **Help** | |  |
| PREV   NEXT | **FRAMES**    **NO FRAMES**     **All Classes** |  |


---


## **Uses of Interface sbrt.shell.io.OutputFile**

| Packages that use OutputFile | |
| --- | --- |
| **sbrt.shell.io** | Provides classes and interfaces for reading and writing files. |
| **sbrt.shell.io.fba** | Provides classes and interfaces for reading and writing files relevant to Flux Balance Analysis. |
| **sbrt.shell.mng.comb** | Provides classes for creating combinatorics process managers. |
| **sbrt.shell.mng.fba** | Provides classes and interfaces for creating Flux Balance Analysis process managers. |
| **sbrt.shell.proc.fba** | Provides classes for converting FBA-related files of different formats. |

Uses of OutputFile in sbrt.shell.io

| Classes in sbrt.shell.io with type parameters of type OutputFile | |
| --- | --- |
| `class` | `MultiOutputFiles<F extends OutputFile<R>,R>`             This class is used to create multiple output files. |

| Subinterfaces of OutputFile in sbrt.shell.io | |
| --- | --- |
| `interface` | `MapOutputFile<K,V>`             This interface is used to represent output files that maps can be written to. |
| `interface` | `MapTextOutputFile<K,V>`             This interface is used to represent output files that maps can be written to as text. |

| Classes in sbrt.shell.io that implement OutputFile | |
| --- | --- |
| `class` | `AbstractMapOutputFile<K,V>`             This abstract class is a skeleton implementation of `MapTextOutputFile`. |
| `class` | `ComoFile<K,V>`             This class is used to write maps to text files in a column-oriented way. |
| `class` | `RomoFile<K,V>`             This class is used to write maps to text files in a row-oriented way. |
| `class` | `SerializedOutputFile<E>`             This class is used to write serialized objects to output files. |
| `class` | `SmoFile<K,V>`             This class is used to write serialized maps to output files. |
| `class` | `TextOutputFile<T>`             This class is used to represent output files containing plain text. |

Uses of OutputFile in sbrt.shell.io.fba

| Subinterfaces of OutputFile in sbrt.shell.io.fba | |
| --- | --- |
| `interface` | `FbaOptOutputFile`             This interface is used to represent FBA optimization output files. |

| Classes in sbrt.shell.io.fba that implement OutputFile | |
| --- | --- |
| `class` | `ColumnOrFbaOptOutputFile`             This class is used to write column-oriented output files containing FBA optimization data. |
| `class` | `RowOrFbaOptOutputFile`             This class is used to write row-oriented output files containing FBA optimization data. |
| `class` | `SerFbaOptOutputFile`             This class is used to write output files containing FBA optimization data as serialized maps. |

Uses of OutputFile in sbrt.shell.mng.comb

| Methods in sbrt.shell.mng.comb that return OutputFile | |
| --- | --- |
| `static OutputFile<java.util.Set<java.lang.String>>` | `SingleElementUnionManager.getOutputFile(ManagerInput<java.lang.String,java.lang.String> input, java.io.PrintWriter out)`             Returns an output file with the name provided in the manager input. |
| `static OutputFile<java.util.Set<java.lang.String>>` | `SingleElementUnionManager.getOutputFile(java.lang.String fileName)`             Returns an output file with the provided name. |
| `static OutputFile<java.util.Set<java.lang.String>>` | `SingleElementUnionManager.getOutputFile(java.lang.String fileName, java.io.PrintWriter out)`             Returns an output file with the provided name. |

Uses of OutputFile in sbrt.shell.mng.fba

| Methods in sbrt.shell.mng.fba that return OutputFile | |
| --- | --- |
| `static OutputFile<java.lang.Double>` | `SvInitialPointManager.getCentralityFile(ManagerInput<java.lang.String,java.lang.String> input, java.io.PrintWriter out)`             Parses and returns the value for `SvInitialPointManager.AVG_P_FILE_NAME` in the provided manager input. |
| `static OutputFile<java.lang.Integer>` | `SvCdHarFluxVectorManager.getObsructedMovesFile(ManagerInput<java.lang.String,java.lang.String> input, java.io.PrintWriter out)`             Returns an output file used to store the number of obstructed moves per flux space. |
| `static OutputFile<java.lang.Integer>` | `SvCdHarFluxVectorManager.getObstructedMovesFile(java.lang.String fileName)`             Returns an output file used to store the number of obstructed moves per flux space. |

Uses of OutputFile in sbrt.shell.proc.fba

| Methods in sbrt.shell.proc.fba with parameters of type OutputFile | |
| --- | --- |
| `void` | `MetatoolFileWriter.writeFile(IrrevRxnFluxome fluxome, OutputFile<java.lang.String> file)`             Writes a metatool input file. |

---


|  |  |  |
| --- | --- | --- |
| **Overview** \| **Package** \| **Class** \| **Use** \| **Tree** \| **Deprecated** \| **Index** \| **Help** | |  |
| PREV   NEXT | **FRAMES**    **NO FRAMES**     **All Classes** |  |


---
